# Impact of the Neglected Tropical Diseases on Human Development in the Organisation of Islamic Cooperation Nations

**DOI:** 10.1371/journal.pntd.0003782

**Published:** 2015-11-25

**Authors:** Peter J. Hotez, Jennifer R. Herricks

**Affiliations:** 1 Department of Pediatrics and Molecular Virology and Microbiology, National School of Tropical Medicine, Baylor College of Medicine, Houston, Texas, United States of America; 2 Sabin Vaccine Institute and Texas Children’s Hospital Center for Vaccine Development, Houston, Texas, United States of America; 3 James A. Baker III Institute for Public Policy, Rice University, Houston, Texas, United States of America; 4 Department of Biology, Baylor University, Waco, Texas, United States of America; New York Blood Center, UNITED STATES

The employment of a new “worm index” of human development, together with additional published health information, confirms the important role neglected tropical diseases (NTDs) play in hindering the advancement of many of the world’s Muslim-majority countries.

**Fig 1:**
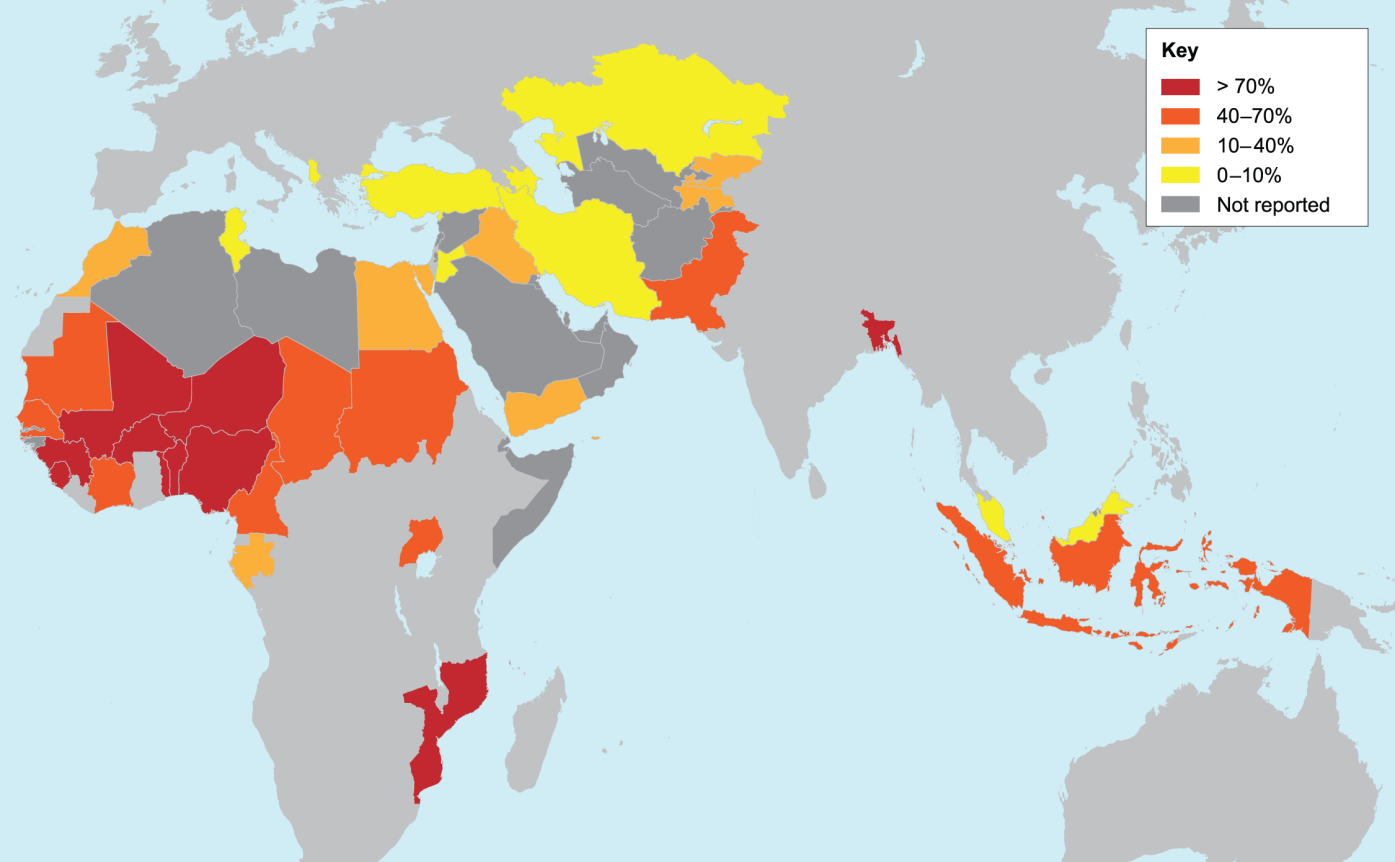
The OIC member nations. Each country is color-coded by the percent of its population that lives on less than US$2 (purchasing power parity [PPP]) per day according to World Bank figures between 2005 and 2013. Suriname and Guyana are not shown.

The Organisation of Islamic Cooperation (OIC, previously the Organisation of the Islamic Conference) is the major inter-governmental organization of 57 Muslim-majority countries, with a mission to promote human rights (especially those of children, women, and the elderly), education, trade, and good governance (Fig 1) [1]. Under the OIC charter, the advancement of science and technology through cooperative research is also a key component [1,2]. In 2009, one of us (PJH) reviewed the available data on the major NTDs and found that many of these diseases disproportionately affected OIC countries, particularly the poorest nations of the Sahel and elsewhere in sub-Saharan Africa and Asia [3]. A previous survey of the 28 largest OIC nations—each with a population of at least 10 million people and comprising more than 90% of the populations of the OIC—found that they accounted for 35%–40% of the world’s soil-transmitted helminth infections and 46% of cases of schistosomiasis, in addition to approximately 20% of the cases of trachoma and leprosy [3]. Given the known impact of these NTDs on both public health and socioeconomic development, it was recommended that scale-up of mass treatment for these diseases should commence in the most affected OIC nations [3]. However, we find that it has been difficult to make progress against poverty and NTDs in the OIC nations.

Today, through support of the United States Agency of International Development’s (USAID) NTD Program, national efforts to control or eliminate NTDs through mass treatments are underway in eight sub-Saharan African OIC countries, in addition to Bangladesh and Indonesia [4]. Efforts are also underway through the NTD Programme of the United Kingdom Department for International Development (UK DFID) [5], while the END Fund, a private philanthropic initiative, supports programs in sub-Saharan African OIC countries, including Nigeria, Niger, and Mali, as well as Yemen, which also receives NTD program support from the World Bank [6].

An updated review and “scorecard” confirm widespread poverty and disease remaining among the 30 most populated OIC countries—those with populations approaching 10 million people or more, and comprising more than 90% of the estimated 1.6 billion people living in these countries (Table 1) [7–9]. At least 40% of the four largest Muslim-majority countries (Indonesia, Pakistan, Nigeria, and Bangladesh) with a combined population of almost 800 million people [7] live on less than US$2 per day [8]. Moreover, while nine of the 30 OIC countries have United Nations Development Programme (UNDP) human development indices (HDIs) in the “high” or “very high” category, 14 are in the “low” category, with some Sahelian OIC nations, such as Burkina Faso, Cameroon, Chad, Mali, Niger, Senegal, and Sudan ranking at or near the bottom of the UNDP’s list of HDIs [9].

**Table 1:** Economic indicators of the 30 most populated OIC countries.

Rank	Country	Population [7]	Living on <US$2 per day [8]	Human Development Index (HDI) [9]	HDI Classification [9]	HDI Rank [9]
1	Indonesia	253 million	43.3% (2011)	0.684	Medium	108
2	Pakistan	185 million	50.7% (2011)	0.537	Low	146
3	Nigeria	179 million	82.2% (2010)	0.504	Low	152
4	Bangladesh	159 million	76.5% (2010)	0.558	Medium	142
5	Egypt	83 million	15.4% (2008)	0.682	Medium	110
6	Iran	78 million	8% (2005)	0.749	High	75
7	Turkey	76 million	2.6% (2011)	0.759	High	69
8	Algeria	40 million	N.R.^a^	0.717	High	93
9	Uganda	39 million	62.9% (2013)	0.484	Low	164
10	Sudan	39 million	44.1% (2009)	0.473	Low	166
11	Iraq	35 million	21.2% (2012)	0.642	Medium	120
12	Morocco	33 million	14.2% (2007)	0.617	Medium	129
13	Afghanistan	31 million	N.R.	0.468	Low	169
14	Malaysia	30 million	2.3% (2009)	0.773	High	62
15	Saudi Arabia	29 million	N.R.	0.836	Very High	34
16	Uzbekistan	29 million	N.R.	0.661	Medium	116
17	Mozambique	26 million	82.5% (2009)	0.393	Low	178
18	Yemen	25 million	37.3% (2005)	0.500	Low	154
19	Cameroon	23 million	53.2% (2007)	0.504	Low	152
20	Syria	22 million	N.R.	0.658	Medium	118
21	Cote d’Ivoire	21 million	59.4% (2008)	0.452	Low	171
22	Niger	19 million	76.1% (2011)	0.337	Low	187
23	Burkina Faso	17 million	72.4% (2009)	0.388	Low	181
24	Kazakhstan	17 million	0.8% (2010)	0.757	High	70
25	Mali	16 million	78.8% (2010)	0.407	Low	176
26	Senegal	15 million	60.3% (2011)	0.485	Low	163
27	Chad	13 million	60.5% (2011)	0.372	Low	184
28	Tunisia	11 million	4.5% (2010)	0.721	High	90
29	Azerbaijan	10 million	2.4% (2008)	0.747	High	76
30	United Arab Emirates	09 million	N.R.	0.827	Very High	40
	Total for OIC all countries	1.56 billion				

a = Not reported between years 2005 and 2013

Based on information from the Preventive Chemotherapy and Control (PCT) database of the World Health Organization (WHO) updated in 2014, helminthic NTDs are still widespread among the OIC countries [10–15]. As shown in Table 2, while the combined population of the 30 most-populated OIC countries of 1.56 billion people accounts for approximately 20% of the global population, it accounts for 37% of school-aged children requiring annual deworming for their intestinal helminth infections [10, 11] and 50% of school-aged children requiring preventive chemotherapy (PC) treatments for schistosomiasis [12, 13]. These OIC nations also account for one-third of the global population requiring PC for lymphatic filariasis (LF) [14, 15].

**Table 2:** The helminthic neglected tropical diseases of OIC countries in 2013.

Country	School-aged children requiring annual deworming for their intestinal helminth infections [10]	School-aged children requiring preventive chemotherapy treatments for schistosomiasis [12]	Populations requiring preventive chemotherapy for lymphatic filariasis [14]	Total helminths (adding three categories)	Worm Index [16]
Indonesia	48.3 million	<0.1 million	99.7 million	148.0 million	0.585
Pakistan	21.2 million	Not endemic	Not endemic	21.2 million	0.115
Nigeria	46.4 million	23.2 million	114.3 million	183.9 million	1.027
Bangladesh	31.8 million	Not endemic	49.7 million	81.5 million	0.513
Egypt	No PC required	0.1 million	0.6 million	0.7 million	0.008
Iran	Not reported	Not endemic	Not endemic	0	0
Turkey	No PC required	Not endemic	Not endemic	0	0
Algeria	Not reported	Not endemic	Not endemic	0	0
Uganda	11.1 million	4.1 million	14.9 million	30.1 million	0.772
Sudan	9.9 million	4.7 million	19.9 million	34.5 million	0.885
Iraq	1.4 million	No PC required	Not endemic	1.4 million	0.040
Morocco	Not reported	Not endemic	Not endemic	0	0
Afghanistan	9.4 million	Not endemic	Not endemic	9.4 million	0.303
Malaysia	No PC required	Not endemic	0.7 million	0.7 million	0.023
Saudi Arabia	Not reported	No PC required	Not endemic	0	0
Uzbekistan	0.3 million	Not endemic	Not endemic	0.3 million	0.010
Mozambique	7.3 million	5.2 million	17.7 million	30.2 million	1.162
Yemen	6.3 million	2.9 million	Undergoing surveillance	9.2 million	0.368
Cameroon	5.9 million	3.6 million	17.1 million	26.6 million	1.157
Syria	Not reported	Not endemic	Not endemic	0	0
Cote d’Ivoire	5.2 million	2.4 million	17.4 million	25.0 million	1.190
Niger	5.2 million	3.0 million	12.6 million	20.8 million	1.095
Burkina Faso	4.7 million	1.8 million	13.1 million	19.6 million	1.153
Kazakhstan	Not reported	Not endemic	Not endemic	0	0
Mali	4.3 million	2.4 million	17.3 million	24.0 million	1.500
Senegal	3.8 million	1.9 million	8.1 million	13.8 million	0.920
Chad	3.7 million	1.8 million	7.3 million	12.8 million	0.985
Tunisia	0.1 million	Not endemic	Not endemic	0.1 million	0.009
Azerbaijan	0.2 million	Not endemic	Not endemic	0.2 million	0.020
United Arab Emirates	Not reported	Not endemic	Not endemic	0	0
Total for OIC all countries	226.5 million	57.1 million	410.4 million	694 million	0.445
Globally	609.5 million in 2012 [11]	114.3 million in 2012[13]	1,241.9 million in 2013 [15]	1,965.7 million	0.270
Percentage in 30 leading OIC countries	37%	50%	33%	35%	

**Fig 2:**
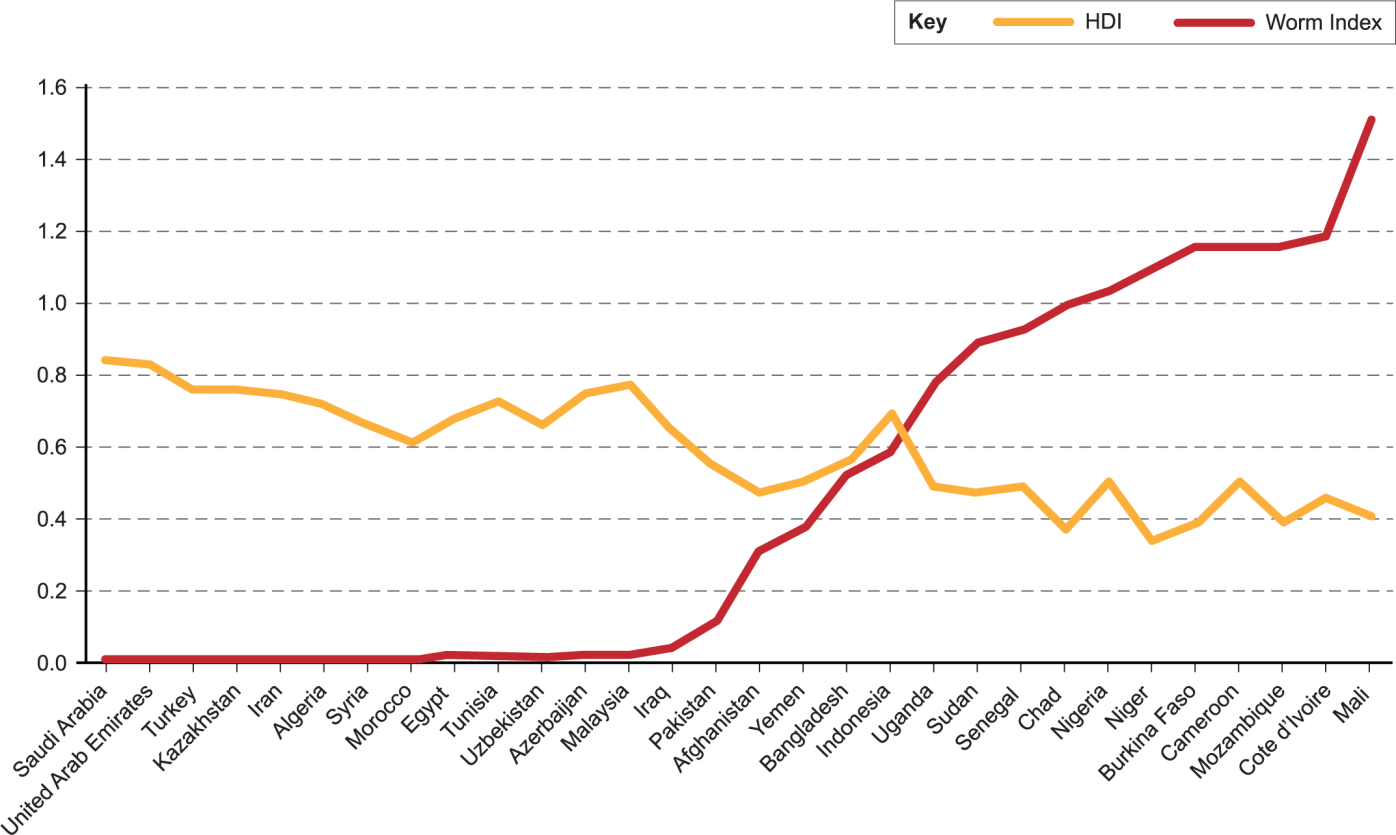
HDI versus worm index of OIC countries (R = -0.85 and *p* < 0.0001).

Such data can be used to calculate a “worm index” of human development in which the number of school-aged children requiring PC for both intestinal helminth and schistosomiasis is added to the population requiring PC for LF, and then divided by the total population of a given nation [16]. It was found previously for the world’s 25 most populated countries that as the HDI fell into the medium range below 0.700, the worm index began to increase sharply towards 0.400; there was a significant rise in the worm index toward 1.000 as the HDI fell into the low range, below 0.500 [16]. As shown in Fig 2, this inverse relationship between HDI and worm index is also true of the OIC countries (R = -0.85 and *p* < 0.0001). Remarkably, none of the OIC countries had an HDI over 0.85. The highest worm indices are among Sahelian nations, followed by other sub-Saharan African countries, and then the large Muslim-majority countries of Asia. Overall, the mean worm index of OIC countries (0.445) was substantially higher than our global estimates (0.270).

The links between intestinal helminth infections and schistosomiasis among school-aged children and human development were summarized previously, and include effects on childhood growth and cognition; similarly, there are links between LF and productive capacity [16].

Beyond helminth infections used to calculate the worm index of human development, as shown in Table 3, the major OIC countries also account for 44% of the global population at risk for onchocerciasis, mostly those in the Sahel and Yemen [17]. Moreover, 68% of the incident cases of cutaneous leishmaniasis (CL) occur in OIC countries, especially those in the Middle East and North Africa [18]. Among the bacterial NTDs, OIC countries account for almost 20% of the world’s registered leprosy cases [19], while trachoma is an important disease in the Sahelian and other OIC countries [20,21]. Of the 30 most populated OIC countries, only 11 are considered by WHO to be non-endemic for trachoma [20,21].

**Table 3:** Other major neglected tropical diseases.

Country	Population at risk for onchocerciasis in 2013 [17]	Incident cases of cutaenous leishmaniasis [18]	Leprosy registered prevalence in 2013 [19]	Trachoma in 2012 [20,21]
Indonesia	0	0	19,730	Non-endemic
Pakistan	0	21,700 to 35,700	657	Endemic
Nigeria	50.1 million	30 to 50	3,626	Endemic
Bangladesh	0	0	3,087	Non-Endemic
Egypt	0	1,300 to 2,200	N.R.^a^	Endemic
Iran	0	69,000 to 113,300	19	Surveillance
Turkey	0	6,900 to 11,300	No information^b^	Non-endemic
Algeria	0	123,300 to 202,600	No information	Endemic
Uganda	4.3 million	0	N.R.	Endemic
Sudan	0.4 million	15,000 to 40,000	1,386	Endemic
Iraq	0	8,300 to 16,500	3	Surveillance
Morocco	0	9,600 to 15,800	37	Surveillance
Afghanistan	0	113,100 to 226,200	44	Endemic
Malaysia	0	0	353	Non-endemic
Saudi Arabia	0	9,600 to 15,800	4	Non-endemic
Uzbekistan	0	710 to 1,400	No information	Non-endemic
Mozambique	0.1 million	0	N.R.	Endemic
Yemen	0^c^	3,000 to 6,000	425	Endemic
Cameroon	8.8 million	280 to 550	419	Endemic
Syria	0	64,100 to 105,300	N.R.	Non-endemic
Cote d’Ivoire	2.3 million	5 to 10	932	Endemic
Niger	No PC required	No data	375	Endemic
Burkina Faso	0.2 million	No data	250	Endemic
Kazakhstan	0	40 to 70	No information	Non-endemic
Mali	5.1 million	290 to 580	276	Endemic
Senegal	0.2 million	40 to 80	404	Endemic
Chad	2.5 million	No data	378	Endemic
Tunisia	0	21,400 to 35,100	0	Non-endemic
Azerbaijan		50 to 80	No information	Non-endemic
United Arab Emirates	0	0	0	Non-endemic
Total for all countries	74.0 million	467,745 to 828,620	32,405	
Globally	169.2 million	690,900 to 1,213,300	180,618	
Percentage in the 30 OIC countries	44%	68%	18%	

^a^ No reported data available

^b^ No information presented by WHO

^c^ There may be fewer than 1 million people at risk in Yemen, but this number is not reported by the WHO

The findings of widespread endemic NTDs, including the major helminth infections, CL, and trachoma, have important implications for the overall development of the world’s most populated OIC countries. In addition to their impact on human development, these NTDs actually promote poverty because of their chronic and debilitating effects, especially on women and children [22,23].

It is important for all nations to respond to diseases of poverty, such as NTDs. In recognition of this, the United Nations has incorporated the elimination of NTDs into their new Sustainable Development Goals. The NTDs are also important because of their potential to emerge or re-emerge in the setting of conflict and post-conflict situations, as we have seen in Africa and the Middle East [24,25]. Therefore, the leadership of the OIC may wish to further emphasize targeting the NTDs for control and elimination, along the lines of the 2012 London Declaration for NTDs and a 2013 World Health Assembly resolution [26,27].

Based in part on a recent survey of experts [28], the control and elimination of NTDs will require both a scale-up of global and integrated mass treatment programs, as well as the advancement of new technologies for NTDs [29]. Given that the charter of the OIC includes scientific cooperation and advancing technologies, such efforts are within its scope. The Islamic Academy of Sciences founded in 1986 could be a key arm for the OIC in this activity [30]. Potential partners include programs such as the US Science Envoy Program, created by the White House and State Department under the Obama administration in order to reach out scientifically to OIC countries through science and vaccine diplomacy, as well as programs like the NTD Support Center, established by the Task Force for Global Health, which works with communities to address the challenges associated with implementing effective NTD elimination strategies [31,32]. Together, such scientific cooperation could produce a new generation of “antipoverty” drugs, diagnostics [33], and vaccines in order to combat the major NTDs now affecting selected OIC countries as well as other nations trapped in poverty.
